# Editorial: Mesenchymal Stromal Cell Therapy for Regenerative Medicine

**DOI:** 10.3389/fncel.2022.932281

**Published:** 2022-05-26

**Authors:** Vivian Capilla-González, Vicente Herranz-Pérez, Rachel Sarabia-Estrada, Nadir Kadri, Guido Moll

**Affiliations:** ^1^Department of Regeneration and Cell Therapy, Andalusian Center of Molecular Biology and Regenerative Medicine (CABIMER)-University of Pablo de Olavide-University of Seville-CSIC, Seville, Spain; ^2^Department of Cell Biology, Functional Biology and Physical Anthropology, School of Biological Sciences, University of Valencia, Valencia, Spain; ^3^Department of Neurologic Surgery, Mayo Clinic Florida, Jacksonville, FL, United States; ^4^Department of Laboratory Medicine, Karolinska Institutet, Stockholm, Sweden; ^5^Science for Life Laboratory (SciLife), Division of Infectious Diseases, Department of Medicine Solna, Karolinska Institutet and Karolinska University Hospital, Stockholm, Sweden; ^6^BIH Center for Regenerative Therapies (BCRT) and Berlin-Brandenburg School for Regenerative Therapies (BSRT), Berlin Institute of Health (BIH) at the Charité Universitätsmedizin Berlin, Corporate Member of Freie Universität Berlin, Humboldt-Universität zu Berlin, Berlin, Germany

**Keywords:** mesenchymal stromal/stem cell (MSC), clinical translation, immunomodulation, regeneration, modes of action (MoA)

## Introduction

**Figure 1:**
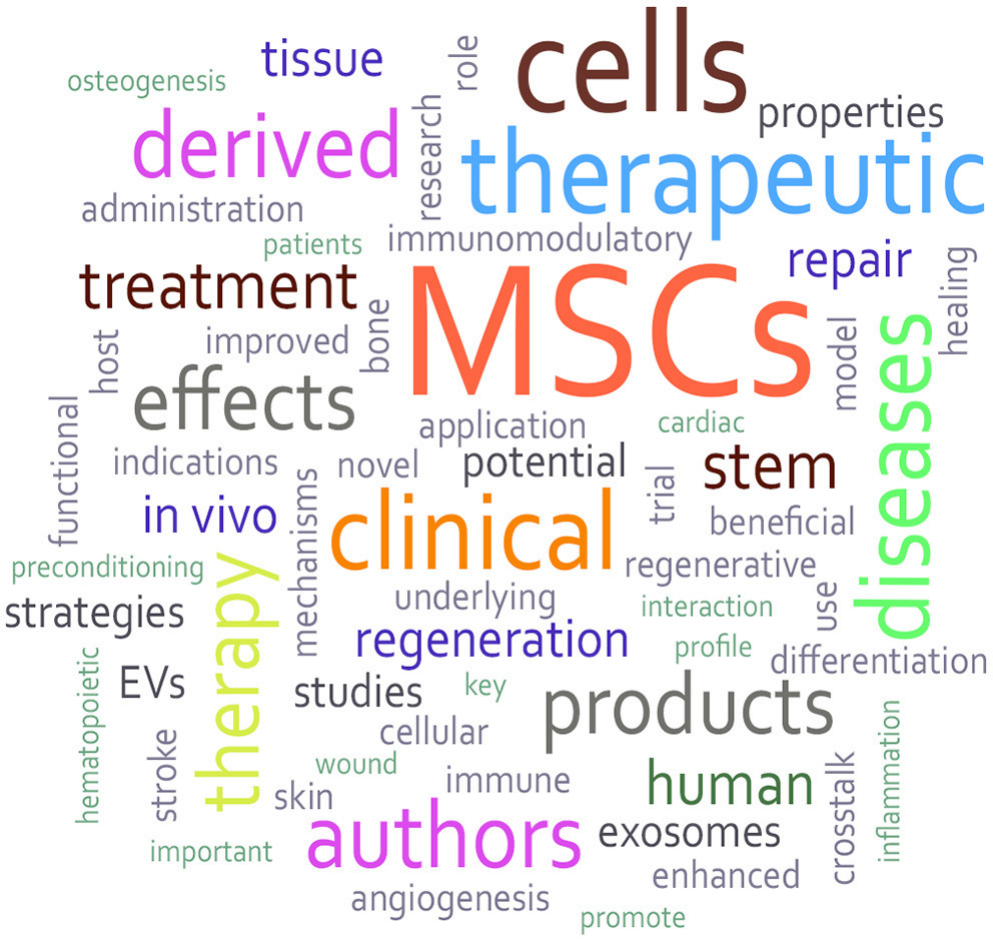
Word cloud of the most frequent terms. This picture describes the most commonly used words in the Editorial on the Research Topic Mesenchymal Stromal Cell Therapy for Regenerative Medicine. Note that the size of the words is proportional to their frequency.

Mesenchymal stromal/stem cell (MSC) therapies are increasingly explored as novel regenerative and immunomodulatory approaches to treat or prevent diseases (Pittenger et al., 2019; Hmadcha et al., 2020; Moll et al., 2020b; Ringdén et al., 2022). These cells exhibit potent paracrine properties that can modulate host immune responses, lower inflammation, and orchestrate endogenous tissue repair, at both the local and the systemic level through multiple pathways (Singer and Caplan, 2011; Doorn et al., 2012). MSCs possess tropism toward damaged and inflamed tissues, where they can engraft short-term and exert their therapeutic effects by both direct and indirect mechanisms (Doorn et al., 2012; Galipeau and Sensebe, 2018; Soria et al., 2019). MSC products can be prepared from multiple sources (Moll et al., 2019, 2022), rapidly expanded and biobanked for clinical application. All these advantages make this cell type a versatile tool in regenerative medicine. The goal of our Research Topic is to highlight the latest advances in applications of MSCs for the treatment of a variety of diseases and their modes of action (MoA). A better understanding of the mechanisms underlying the therapeutic effect of MSCs can provide crucial insight into innovative strategies to enhance their effectiveness in clinical application (Singer and Caplan, 2011; Doorn et al., 2012; Galipeau and Sensebe, 2018; Moll et al., 2019, 2020b, 2022; Pittenger et al., 2019; Ringdén et al., 2022). The subjects covered within this Research Topic include: (a) Therapeutic application of MSCs for major clinical indications, (b) Cellular and molecular mechanisms underlying therapeutic effects of MSCs, and (c) Strategies for enhancement of the therapeutic effects of MSCs and their products. Here, we summarize the 37 manuscripts that were submitted to this Research Topic (Figure 1).

### Therapeutic Application of MSCs for Major Clinical Indications

Numerous studies have assessed MSC therapies for treating a wide array of pathologies owing to their potent immunomodulatory and regenerative properties (Kabat et al., 2020). Typical clinical indications for MSC therapy include musculoskeletal repair, neurological and cardiovascular pathologies (e.g., stroke), cancer treatment, hematological and auto- or allo-immune complications (e.g., graft-vs.-host disease, GvHD), and more recently also complications associated with coronavirus disease 2019 (COVID-19), such as acute respiratory distress syndrome (ARDS) and sepsis (Soria-Juan et al., 2019; Hmadcha et al., 2020; Ringdén et al., 2022).

Merimi et al. and Sagaradze et al. give a general introduction on MSC therapy in their review articles “*The therapeutic potential of MSCs for regenerative medicine: current knowledge and future understandings*” and “*MSCs as critical contributors in tissue regeneration*.” The authors here introduce important aspects of MSC therapy, such as the origin, characteristics and definition of MSCs, the prominent MSC product diversification in recent years, key therapeutic applications, clinical indications and considerations on their use, including mechanistic and functional aspects of MSC therapeutics and key mediators.

Crippa et al. from the “IRCCS San Raffaele” in Milano, Italy, elucidate the “*Role of ex vivo expanded MSCs in determining hematopoietic stem cell transplantation outcome*.” The transplantation of hematopoietic stem and progenitor cells (HSPC/HSCT) is the only curative option available today for several hematological and non-hematological diseases. However, HSCT is associated with several early and late complications, such as low HSPC engraftment and impaired hematopoietic recovery, immune-mediated graft rejection, and GvHD in the case of allotransplantation (Ringdén et al., 2022). MSCs are a key structural and functional component of the hematopoietic bone marrow niche (Sacchetti et al., 2007) and the authors here summarize the intrinsic supportive role of MSCs *in vivo* and the adoptive transfer of *ex vivo*-expanded cells.

In their review article, Rascón-Ramírez et al. from the “Hospital Clinico San Carlos” in Madrid, Spain, were asking: “*Are we ready for cell therapy to treat stroke?*” The authors evaluated clinical trials using cell therapy to treat stroke and their limitations from the year 2000 onwards. The authors found that stem cell treatment is safe with only transient mild adverse events, with considerable variation in clinical trials in terms of statistical design, sample size, the cells used, the routes of administration, and the functional assessments, thus making it difficult to compare studies. The authors are calling for improvements in the experimental protocol as a main element to improve future studies and more standardization in clinical trials in order to aid comparison. Furthermore, considering a non-invasive administration route would be a critical point for neurological diseases. In this context, intranasal delivery of MSCs was shown to be safe and effective (Soria et al., 2019; Aguilera et al., 2021), bringing a promising therapeutic alternative.

Due to the perivascular nature of MSCs surrounding and maintaining the integrity and function of blood vessels (Bianco et al., 2008, 2013; Crisan et al., 2008), lung pathology, such as the rapidly progressing COVID-19 associated ARDS (Moll et al., 2020a), but also the more chronic idiopathic pulmonary fibrosis (IPF) (Meltzer and Noble, 2008), are among frequently envisioned indications for MSC therapy (Ringdén et al., 2022). Kassem and Kamal from “The British University in Cairo,” Egypt, give an update on “*MSCs and their extracellular vesicles (EVs): a potential game changer for the COVID-19 crisis*,” to employ their immunomodulatory and regenerative properties for lung repair. In turn, Yang et al. from the “Center for Respiratory Medicine” at the China-Japan Friendship Hospital in Beijing, China, review “*Therapeutic applications of MSCs in IPF*.” The authors present an in-depth analysis of relevant factors for the optimization of MSC treatment, including the use of MSCs from different sources for lung repair, different administration routes, timing, dosing, frequency, and potential pretreatment of the clinical MSC products, to guide the design of future clinical research and to identify novel therapeutic options for these complex diseases.

Bruno et al. from the “University of Torino”, Italy, give an overview on: “*Human liver stem cells (HLSCs): a liver-derived MSC-like population with pro-regenerative properties*.” These HLSCs were first described in 2006 as a new stem cell population obtained from the highly vascularized healthy human livers (Herrera et al., 2006), which possess—similar to MSCs—multipotency and immunomodulatory properties. The HLSCs were shown to contribute to tissue repair and regeneration in different *in vivo* models, leading to >5 granted patents and >15 peer-reviewed scientific articles. The cells are now employed in early clinical trials to evaluate their safety post intrahepatic injection in infants with inherited neonatal onset hyperammonemia.

The skin is the largest organ of the human body and skin injuries can damage this important barrier. Three review articles were contributed to our topic from researchers at the “Andalusian Network of Design and Translation of Advanced Therapies” at “Virgen de las Nieves University Hospital” in Granada, Spain. These reviews give an excellent overview on the treatment of skin disease with “cell products” (i.e., MSC) and “cell-conditioned” media (CCM), as well as “MSC-derived exosomes” in dermatology. Sierra-Sánchez et al. report in their review “*Current advanced therapies based on human MSCs for skin diseases*” that in total 13 types of MSC products have been used as advanced therapies in dermatology in the past 5 years (2015–2020). The most studied MSC types were isolated from umbilical cord (UC-MSCs), adipose tissue (AT-MSCs) and bone marrow (BM-MSCs). The most studied diseases were wounds, ulcers, burns and psoriasis. Montero-Vilchez et al. report in their article “*MSC-conditioned medium for skin diseases: a systematic review*” that MSC-CCM improved wound healing, hair restoration, skin rejuvenation, atopic dermatitis, and psoriasis, in both animal models and humans, and decreased hypertrophic scars and flap ischemia in animal models, with further studies needed to standardize CCM manufacturing. In addition, Quiñones-Vico et al. reviewed “*The role of exosomes derived from MSCs in dermatology*” for both *in vitro* and *in vivo* models of different skin conditions.

In some cases, patients present a life-threatening condition or serious disease that requires the urgent access to investigational medical product (e.g., MSC products). For those cases, the U.S. Food and Drug Administration (FDA) may authorize expanded access, to gain access to an investigational medical product for treatment outside of clinical trials. Khan et al. from “The Interdisciplinary Stem Cell Institute” at the University of Miami share their experiences in their article: “*The Interdisciplinary Stem Cell Institute's Use of Food and Drug Administration-Expanded Access Guidelines to Provide Experimental Cell Therapy to Patients With Rare Serious Diseases*.”

### Cellular and Molecular Mechanisms Underlying Therapeutic Effects of MSCs

An in-depth understanding of the molecular mechanisms underlying MSCs' beneficial therapeutic effects in different clinical indications is essential to manufacture safe and effective therapies tailored to the individual patient need (Moll et al., 2020b). This entails multifaceted pleiotropic effects of MSCs in immunomodulation and tissue regeneration specific to the cell product (Moll and Le Blanc, 2015; Moll et al., 2016, 2019, 2020a,b, 2022; Giri and Moll, 2022), but also direct and indirect crosstalk between therapeutic cells and the host cells, that substantially depends on the competence of the host to respond to the treatment (Escacena et al., 2015; Capilla-Gonzalez et al., 2018; Moll et al., 2020b; Galipeau, 2021; Galipeau et al., 2021; Krampera and Le Blanc, 2021).

Müller et al. from the “Technical University in Dresden,” Germany, give and update on the “*Immunomodulatory Properties of MSCs*.” The authors here elucidate how MSCs modulate the phenotype and functional properties of various immune cells of both, the adaptive and innate branches of the immune system, that play an important role in the pathogenesis of inflammatory disorders. Macrophages are at the nexus of MSC immunomodulation and clinical potency (Galipeau, 2021). Lu et al. from “The Biotherapy Center” at Sun Yat-sen University in Guangzhou, China, review in detail the “*MSC-Macrophage crosstalk and maintenance of inflammatory microenvironmental homeostasis*.” The authors' major focus is on the interaction between MSCs and macrophages, such as “cell-to-cell contact,” “soluble factor secretion,” and “organelle transfer,” but also the role of “MSC-macrophage crosstalk” in the development of pathology and maintenance of homeostasis of inflammatory microenvironments, and “strategies to optimal application” in immune-related treatments.

Another key axis in endogenous repair is the modulation of angiogenesis and blood circulating endothelial cells (CECs) and endothelial progenitor cells (EPCs) (Farinacci et al., 2019). Bouland et al. from the Free University of Brussels in Belgium elucidate the “*Crosstalk between MSCs and EPCs in bone regeneration*.” The authors emphasize that bone regeneration is a complex but well-orchestrated process, based on the interaction between osteogenesis and angiogenesis in both the physiological and pathological setting, with specific conditions requiring additional support. Here, MSC-EPC cocultures have shown some synergy, with EPCs fostering osteogenesis, while MSCs promote both angiogenesis and osteogenesis, thereby boosting bone healing. Thus, novel therapeutic strategies aiming to employ this synergy are of great interest in bone repair, but may also have to take into account any potential functional loss of autologous MSC competence during disease progression (Escacena et al., 2015; Capilla-Gonzalez et al., 2018; Andrzejewska et al., 2019).

#### Extracellular Vesicles as Mediators of MSCs Therapeutic Activity

The production and release of EVs, including exosomes, from MSCs is perceived to be a major mediator of their therapeutic effects for several pathological conditions (Lener et al., 2015). Using a hippocampal penetrating brain injury mouse model to analyze kinematic changes, León-Moreno et al. found a significant decrease in locomotion speed in both open field and tunnel walk tests, but locomotion speed and displacement pattern preservation following intranasal administration of endometrial (EM)-MSC-derived EVs, thus suggesting that the EVs confer neuroprotection to the damaged hippocampus. De Luna et al. reviewed the role of “*MSC-derived EVs as silver linings for cartilage regeneration*.” Ghafouri-Fard et al. summarize “*The emerging role of exosomes in the treatment of human disorders with a special focus on MSC-derived Exosomes*.” Zhong et al. review “The emerging potential of exosomes on adipogenic differentiation MSCs”. Zhang J. et al. review the “*Potential networks regulated by MSCs in acute-on-chronic liver failure (ACLF): exosomal miRNAs and intracellular target genes*,” identifying beneficial effects of MSC-derived exosomes. Zhang Y. et al. investigated the therapeutic potential of exosomes derived from amniotic fluid stem cells in a rat model of skin wound repair. They found accelerated wound healing and improved regeneration of hair follicles, nerves, vessels, and increased proliferation of cutaneous cells, and more natural distribution of collagen during wound healing, while preventing excessive aggregation of myofibroblasts and the extracellular matrix. Lai et al. found that AT-MSC-derived exosomes decreased cardiomyocyte apoptosis and hypertrophy in the heart of mice with myocardial ischemia/reperfusion injury, concluding that they act as potent cardioprotectors.

#### Exemplary Studies on Cellular Crosstalk of MSCs With Their *in vivo* Environment

Satani et al. from the “McGovern Medical School at UTHealth” in Houston, Texas, studied how “*A combination of atorvastatin and aspirin enhances the pro-regenerative interactions of BM-MSCs and stroke-derived monocytes in vitro*.” They found that these two most commonly prescribed stroke medications (statin and statin plus aspirin) influence the secretome of MSCs and their interaction with monocytes from stroke patients to promote the beneficial antiinflammatory effect of MSCs. In turn, Maisonneuve et al. shed light on how human dental pulp derived stromal cells (DPSCs) react toward interaction with *Streptococcus mutans*, which is involved in dental pulp necrosis and cardiovascular tissue infections. In response to infection, DPSCs adopted a proinflammatory profile, strengthening the establishment of the dental pulp inflammation.

### Strategies to Enhance the Therapeutic Effects of MSCs and Their Products

Recent research efforts focus on strategies for further improving the therapeutic efficacy and safety of MSCs and their products, thus helping to achieve better treatment outcomes. These entail amongst others improved *in vivo* characterization and cell sorting, preconditioning of MSCs, cellular engineering and the use of structural supports/3D systems.

#### Improved *in vivo* Characterization of Cell Sources and Cell Sorting

Valiuliene et al. from the “Life Sciences Center” at Vilnius University in Lithuania studied the “*Metabolic profile and neurogenic potential of human amniotic fluid MSC-like stem cells from normal versus fetus-affected gestations*.” The authors found some characteristic differences in surface markers, phosphorylation rate, ATP, ROS, and cytokine secretion between the two groups, which may indicate metabolic and phenotypic differences that need to be considered in their clinical use. Xie et al. from Sun Yat-sen University in Guangzhou, China, studied how “*Cardiac-derived CD51-positive MSCs enhance cardiac repair through SCF-mediated angiogenesis in mice with myocardial infarction*.” Most vascularized tissues contain resident MSCs, but there is no typical marker to identify resident cardiac MSCs, and the authors here identify CD51 as a novel marker for cardiac resident MSCs.

#### Preconditioning of MSCs to Improve Their Functionality

An injured tissue constitutes an ischemic and hypoxic environment that damages the implanted therapeutic cells, leading to apoptosis and thus compromising their capacity in the early stages of cell transplantation. Zhao et al. demonstrate that hypoxic culture preconditioning may constitute a promising strategy to enhance cellular viability and angiogenesis of transplanted AT-MSCs. Lucciola et al. have studied the “*Impact of sustained TGF-*β*-receptor inhibition on chromatin accessibility and gene expression in culture human endometrial EM-MSCs*.” The authors found that A83-01, a selective TGF-β-receptor inhibitor, promotes the expansion of EM-MSCs in culture by blocking differentiation and senescence, with in-depth analysis of the underlying gene networks and genome-wide chromatin changes. Bai et al. report how “*Glycyrrhizic acid promotes osteogenic differentiation of human BM-MSCs by activating the Wnt/*β*-catenin signaling pathway*,” thus indicating the potential of pharmacological pretreatment for improvements in bone repair. Preconditioning of MSCs also modifies the content and secretion of EVs. Peltzer et al. present that “*IFN-*γ *and hypoxia priming have limited effect on the miRNA landscape of human MSC-derived EVs*.” Li et al. found that “*miRNA-27a-5p is abundant in small EVs derived from epimedium (EPI)-pre-conditioned BM-MSCs stimulate osteogenesis by targeting Atg4B-mediated autophagy*.” Feng et al. reviewed the “*Effect of melatonin for regulating MSCs and their EVs*” in preclinical and clinical studies.

#### Cellular Engineering to Improve MSC Function

Garcia-Bernal et al. from Murcia in Spain studied how “*Exofucosylation of AT-MSCs alters their secretome profile*.” The authors found that the exofucosylation modification that improves MSC *in vivo* trafficking also improved their secretome to promote their antiinflammatory properties, which could be beneficial in treatment of autoimmune, inflammatory, and degenerative diseases. Pawitan et al. reviewed the “*Enhancement of the therapeutic capacity of MSCs by genetic modification*.” Of the 85 articles reviewed, 51 studies focused tumor/cancer/metastasis, while 34 studies focused on non-cancerous pathologies. In line, Han et al. studied how “*Knockdown of POSTN inhibits osteogenic differentiation of MSCs from patients with steroid-induced osteonecrosis*,” while Zhang W. et al. studied how “*Upregulation of PARKIN accelerates osteoblastic differentiation of BM-MSCs and bone regeneration by enhancing autophagy and* β*-catenin signaling*.”

#### Structural Supports and 3D Environment for MSCs

Among the strategies to preserve MSC biological functions are those mimicking the natural habitat of MSCs during cell therapy. Lin et al. from “Tsing Hua University” in Taiwan demonstrate how “*3D-spheroids of UC-MSC-derived Schwann Cells (SCs) promote peripheral nerve regeneration*.” The authors used a well-defined non-genetic approach to phenotypically, epigenetically, and functionally convert UC-MSCs into SC-like cells that simulated sprouting of neurites from neuronal cells. To enhance their therapeutic effectiveness, the authors assembled the cells in 3D spheroids with a marked increase in their neurotropic, proangiogenic and anti-apoptotic profile. Marinaro et al. from Cáceres in Spain tested “*Laparoscopy for the treatment of congenital hernia: Use of surgical meshes and MSCs in a clinically relevant animal model*.” The authors combined laparoscopy and stem cells incorporated into a mesh by using a fibrin-sealant solution for the treatment of hernia in a swine model, with improved *in vivo* performance in the MSC containing group compared to cell-free mesh.

## Conclusion

This multidisciplinary Research Topic has brought together specialists in Stem Cells, Immunology, Bioengineering, and Cellular Neuroscience, to share their knowledge on current therapeutic strategies and the mechanisms underlying the immune modulation and tissue regeneration that are orchestrated by MSCs. The different articles contained in this Research Topic reflect the great interest, knowledge and innovation in these fields. Here, we have given a summary on the latest clinical developments, efforts to refine the understanding on the MoA of MSC therapeutics, and introduced novel strategies to enhance the efficacy of MSCs and their derived products. Importantly, inflammation and the regulation thereof is an essential part of the healing and regenerative cascades in the human body. The modulation of inflammation is an important aspect of MSCs beneficial properties in the healing of tissue injury or suppression of tissue damage. The many studies contained in this Research Topic indicate that MSCs employ a host of mediators and mechanisms to achieve this goal, with great promise for clinical use.

## Author Contributions

All authors listed have made a substantial, direct, and intellectual contribution to the work and approved it for publication.

## Funding

VC-G receives support of the Consejería de Transformación Económica, Industria, Conocimiento y Universidades co-funded by Fondos FEDER (PY20/00481), the Institute of Health Carlos III co-funded by Fondos FEDER (CP19/00046 and PI20/00341), the Fundación Científica de la Asociación Española Contra el Cáncer (IDEAS20051CAPI), and the Asociación Pablo Ugarte (+VIDA project). VH-P receives funding from the Spanish Ministry of Science, Innovation and Universities (PCI2018-093062) and the Valencian Council for Innovation, Universities Science and Digital Society (PROMETEO/2019/075). Contributions from GM were made possible by the German Research Foundation (DFG) and the German Federal Ministry of Education and Research (BMBF) through the BSRT (GSC203) and BCRT, respectively, and in part supported by the European Union's Horizon 2020 Research and Innovation Program (Horizon 2020 Framework Program) under the Grant Agreements No. 733006 (PACE) and No. 779293 (HIPGEN).

## Conflict of Interest

The authors declare that the research was conducted in the absence of any commercial or financial relationships that could be construed as a potential conflict of interest.

## Publisher's Note

All claims expressed in this article are solely those of the authors and do not necessarily represent those of their affiliated organizations, or those of the publisher, the editors and the reviewers. Any product that may be evaluated in this article, or claim that may be made by its manufacturer, is not guaranteed or endorsed by the publisher.
